# Lung clearance index and diffusion capacity for CO to detect early functional pulmonary impairment in children with rheumatic diseases

**DOI:** 10.1186/s12969-021-00509-1

**Published:** 2021-03-06

**Authors:** Julia Hildebrandt, Anja Rahn, Anja Kessler, Fabian Speth, Dagmar-Christiane Fischer, Manfred Ballmann

**Affiliations:** Department of Pediatrics, Rostock University Medical Centre, Ernst-Heydemann Strasse 8, DE 18057 Rostock, Germany

**Keywords:** Pulmonary impairment, Lung clearance index, Diffusion capacity for carbon monoxide, Immunosuppressive therapy, Children, Rheumatic disease, Juvenile idiopathic arthritis

## Abstract

**Background:**

In adults with rheumatic diseases pulmonary complications are relevant contributors to morbidity and mortality. In these patients diffusion capacity for CO (DLCO) is an established method to detect early pulmonary impairment. Pilot studies using DLCO indicate that early functional pulmonary impairment is present even in children with rheumatic disease albeit not detectable by spirometry and without clinical signs of pulmonary disease.

Since the lung clearance index (LCI) is also a non-invasive, feasible and established method to detect early functional pulmonary impairment especially in children and because it requires less cooperation (tidal breathing), we compared LCI versus DLCO (forced breathing and breath-holding manoeuvre) in children with rheumatic diseases.

**Findings:**

Nineteen patients (age 9–17 years) with rheumatic disease and no clinical signs of pulmonary disease successfully completed LCI and DLCO during annual check-up. In 2 patients LCI and DLCO were within physiological limits. By contrast, elevated LCI combined with physiological results for DLCO were seen in 8 patients and in 9 patients both, the LCI and DLCO indicate early functional pulmonary changes. Overall, LCI was more sensitive than DLCO to detect early functional pulmonary impairment (*p* = 0.0128).

**Conclusions:**

Our findings suggest that early functional pulmonary impairment is already present in children with rheumatic diseases. LCI is a very feasible and non-invasive alternative for detection of early functional pulmonary impairment in children. It is more sensitive and less cooperation dependent than DLCO. Therefore, we suggest to integrate LCI in routine follow-up of rheumatic diseases in children.

## Introduction

Adults with rheumatic diseases are at risk to develop pulmonary impairment secondary to the disease-related chronic inflammation and fibrosis, and/or to a lesser extent due to immunosuppressive therapy. During follow-up the DLCO (CO specific diffusion capacity of the lung) has been shown to be a more sensitive marker for early pulmonary impairment than spirometry [1].

By contrast, this issue has rarely been investigated in pediatric patients with rheumatic diseases, most probably due to the fact that breathing manoeuvre required for DLCO (forced breathing and breath-holding for a mandatory length of time) are challenging. Two recently published studies on children and young adults with juvenile idiopathic arthritis (JIA) with no clinical signs of pulmonary disease and healthy controls stated a significant reduction in DLCO in JIA patients, while spirometry data were fairly comparable between groups. This data indicates, that even pediatric JIA patients are prone to early functional pulmonary impairment not detectable by spirometry [2, 3].

Overall, data on the incidence and prevalence of lung impairment in children with JIA is scarce. Symptoms of early pulmonary impairment, e.g. shortness of breath, dry cough, chest tightness or sleep disturbance, are frequently missing in children with rheumatic diseases. Therefore, pulmonary function testing within the frame of a regular outpatient screening has been recommended [4].

In a healthy lung distribution of inhaled gases is largely homogenous. Pulmonary changes are likely to start in peripheral airways and will increase ventilation heterogeneity. Peripheral airways (diameter < 2 mm) represent 95% of total lung volume, but account for only 10–20% of airflow and resistance as reported by spirometry. Therefore, spirometry is rather insensitive for detection of early peripheral airway impairment [5]. Thus, by the time pulmonary pathologies are symptomatic or detectable by spirometry a reasonable part of the functionally most relevant pulmonary tissue is already affected.

Over the last decades, measurement of ventilation inhomogeneity (LCI) became a well-established screening method for early pulmonary impairment in pediatric patients with underlying pulmonary diseases, e.g. cystic fibrosis (CF) or bronchopulmonary dysplasia (BPD). LCI requires tidal breathing only and turned out to be very feasible in children [6–9]. In CF patients, high resolution computed tomography (HRCT) and LCI have been shown to be equally well suited to detect early structural and/or functional lung diseases and to be more sensitive than spirometry [6].

To investigate possible early functional pulmonary impairment in children with rheumatic diseases, we choose a non-invasive method (LCI) with minimal cooperation needed and compared this to the most sensitive pulmonary function measurement (DLCO) used in adults with rheumatic diseases which implies a rather challenging breathing manoeuvre.

## Methods

Pulmonary functions tests (PFT) were performed by specialized personnel. DLCO and LCI were added to the annual outpatient check-up for pediatric patients with rheumatic disease. Data were collected prospectively. Patients with pulmonary disease like asthma, BPD or acute respiratory infection were excluded.

LCI (nitrogen multiple breath washout) and DLCO (single breath washout with Helium and CO as tracer gases) were measured by means of the EasyOne ProLab (ndd Medical Technologies, Zurich, Switzerland) at the same day. According to American Thoracic Society (ATS) and European Respiratory Society (ERS) standards patients were seated in upright and relaxed position, wearing a nose clip, and breathing through a mouthpiece. To increase compliance the patient could e.g. watch a video. At least two technically acceptable measurements were required to judge the examination as valid [10, 11].

The LCI is defined as the cumulative expired volume required to wash out a tracer gas (e.g. Nitrogen) to 1/40th of the starting concentration, divided by the functional residual capacity.

Multiple studies on healthy people have shown that measurement of the LCI is highly reliable [7, 9]. Depending on devices [12] and tracer gas [13] the upper limit of normal (ULN) of LCI in the pediatric age group is in the range of 7.0–7.7 [8, 9, 14, 15]. In the present study and in accordance with Fuchs et al. [9] the ULN for LCI was set to 7.0.

The lower limit of normal percentage predicted value for DLCO corrected for haemoglobin was set to 80% [16]. The total lung capacity (TLC) was derived from DLCO evaluation as a global lung function parameter. Statistical analysis was performed with Fisher Exact Test because of small sample size as a 2 × 2 contingency table with the categories and groups LCI and DLCO, and pathological and non-pathological result, respectively, using IBM SPSS Statistics 25®. The project was approved by the Ethics Committee University Medicine Rostock (registration number A 2016–0193).

## Results

Thus far, 19 patients (12 girls) suffering from rheumatic disease, age 9–17 years, with no clinical signs of pulmonary disease successfully completed LCI and DLCO during the annual outpatient check-up. Disease activity was evaluated clinically. We have HRCT results for one patient performed prior to this study. None of the patients did a 6-min walk test.

**Fig. 1:**
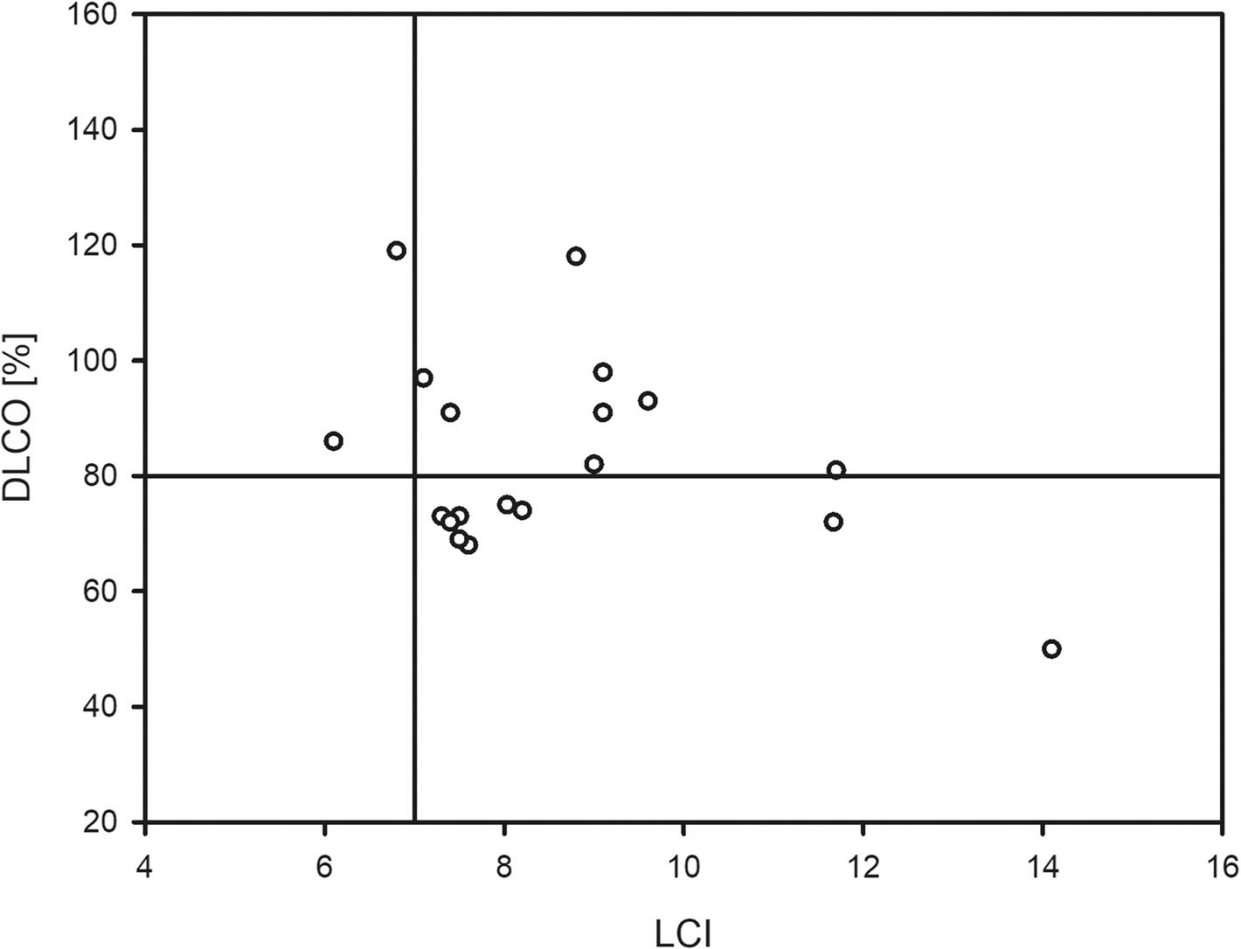
Results from LCI and DLCO in pediatric patients with rheumatic diseases. The lower (DLCO) and upper (LCI) limit of normal are indicated as horizontal and vertical lines, respectively

Clinical data and the results of LCI, DLCO and TLC are summarized in Table 1. In 2 patients LCI and DLCO were within physiological limits. By contrast, elevated LCI combined with physiological results for DLCO were seen in 8 patients and in 9 patients LCI and DLCO indicate early functional pulmonary impairment (Fig. 1).

**Table 1:** Clinical characteristics of patients, data is given as median and range

Number of patients, *n*	19
male/female	7/12
Age [years]	14.0 (9–17)
Duration of disease [years]	4.0 (0–10)
Diagnosis, *n*	
JIA	12
- psoriatic arthritis	*1*
- polyarthritis	*5*
- oligoarthritis	*4*
- undifferentiated JIA	*1*
- enthesitis associated arthritis	*1*
other rheumatic diseases	7
- mixed connective tissue disease	*1*
- juvenile dermatomyositis	*1*
- systemic autoimmune disorders of unspecified identity	*3*
- chronic recurrent multifocal osteomyelitis	*1*
- COPA-Syndrome	*1*
Disease activity, *n*	
active/inactive	10/9
Therapy, ever, *n*	
Methotrexate/Biologicals/None	15/6/3
LCI	8.03 (6.1–14.1)
DLCO [% Hb corrected]	81.0 (50–119)
TLC [% predicted]	91 (56–119)

Data are given as median and range

*JIA* juvenile idiopathic arthritis, *LCI* lung clearance index, *DLCO* diffusion capacity of carbon monoxide, *TLC* total lung capacity

There was one patient with mixed connective tissue disease (LCI 8.03, DLCO 75%) and one patient with juvenile dermatomyositis (LCI 9.0, DLCO 82%). One patient suffered from seropositive polyarthritis and associated vasculitis. Final diagnosis was COPA-Syndrome with signs of granulomatous lung fibrosis in HRCT prior to this study. Subjectively free of pulmonary symptoms this patient showed concordant results in LCI (14.1), DLCO (50%) and spirometry (FEV1 62%, FVC 63%, TLC 56%).

Median (range) TLC was 91 (56–119) % predicted. Median within test variability in this study was 3.65 (0.5–10.9) % in LCI and 5.55 (0.3–13.0) % in DLCO. Thus, within normal limits of ERS/ATS standards [10, 11, 16, 17].

Fisher Exact Test indicates that LCI is a more sensitive parameter than DLCO to detect early pulmonary impairment in pediatric patients with rheumatic diseases (*p* = 0.0128).

## Discussion

Our data confirmed that early functional pulmonary impairment can occur in pediatric patients with rheumatic diseases [2, 3] even in the absence of pulmonary symptoms. While the median DLCO was still within physiological limits, LCI results point to a considerably higher incidence of early functional airway involvement.

Although rheumatic diseases are heterogeneous in terms of the underlying pathophysiology, activation of autoinflammatory and autoimmune processes are inevitably present and may trigger pulmonary impairment [2–4].

DLCO and LCI are especially suited to detect early functional pulmonary changes when spirometry is still within the normal range [2, 3, 6].

Attanasi et al. investigated DLCO in pediatric JIA patients without pulmonary symptoms and stated that reduction in DLCO suggests “abnormalities of the alveolar-capillary interface, compatible with diffuse vascular and/or parenchymal lung disease” [2].

A study in patients with idiopathic pulmonary fibrosis and pulmonary damage detectable by spirometry (median FVC 58%, median FEV1 64%) reported concordant results in median DLCO (41%) and LCI (11.6), as well as concomitant structural airway damage (HRCT) and clinical disease severity [18]. Since none of our patient showed clinical signs of pulmonary disease, the results of this and our study are hardly comparable.

LCI has been shown to be similarly sensitive than HRCT for detection of early pulmonary changes in pediatric CF patients with normal lung function by spirometry [6, 19].

However, LCI is not a linear measure of functional pulmonary impairment. Instead, it is most sensitive for detection of early pulmonary impairment [6]. Advanced and end-stage lung disease is still documented nicely by spirometry [18].

CT-imaging was not yet indicated due to pediatric radiation protection because, except in one patient, there were no signs of pulmonary disease. This patient received imaging during diagnostic work-up prior to this study. Furthermore, the primary focus of this study was on the detection of early functional pulmonary changes rather than the detection of structural aberrations.

Thus, we limited ourselves to LCI and DLCO as tools for routine screening of patients suspicious to suffer from early functional pulmonary impairment [1–4].

The rather small number of patients and the assessment of LCI and DLCO only once during annual check-up must be considered limitations of our study. Similarly, we cannot calculate the correlation between markers of early functional pulmonary changes, underlying disease, disease activity and duration, and/or the influence of immunosuppressive therapy.

Longitudinal studies on larger groups of patients will help to decipher these aspects and identify patients at risk for early pulmonary impairment.

Although, lung involvement in rheumatic disease in children is described with low frequency most authors recommend interdisciplinary routine follow-up for early pulmonary changes even in asymptomatic patients [2–4].

In conclusion, this cross-sectional study showed for the first time that LCI is more sensitive than DLCO for detection of early functional pulmonary changes. Furthermore, we confirmed that such changes are already present in children with rheumatic disease even without clinical signs of pulmonary disease. Therefore, we suggest to integrate LCI in the routine follow-up of children with rheumatic disease.

## Data Availability

The dataset used and analysed in the current study are available from the corresponding author on reasonable request.

## Abbreviations

ATS: American Thoracic Society
BPD: Bronchopulmonary dysplasia
CF: Cystic fibrosis
DLCO: Diffusion capacity for CO
ERS: European Respiratory Society
FEV1: Forced 1 s capacity
FVC: Forced vital capacity
HRCT: High resolution computed tomography
JIA: Juvenile idiopathic arthritis
LCI: Lung clearance index
TLC: Total lung capacity

## References

[1] Spagnolo P, Lee JS, Sverzellati N, Rossi G, Cottin V (2018). The lung in rheumatoid arthritis: focus on interstitial lung disease. Arthritis Rheumatol.

[2] Attanasi M, Lucantoni M, Rapino D, Petrosino MI, Marsili M, Gasparroni G (2019). Lung function in children with juvenile idiopathic arthritis: a cross-sectional analysis. Pediatr Pulmonol.

[3] Pelucchi A, Lomater C, Gerloni V, Foresi A, Fantini F, Marazzini L (1994). Lung function and diffusing capacity for carbon monoxide in patients with juvenile chronic arthritis: effect of disease activity and low dose methotrexate therapy. Clin Exp Rheumatol.

[4] Richardson AE, Warrier K, Vyas H (2016). Respiratory complications of the rheumatological diseases in childhood. Arch Dis Child.

[5] Macklem PT (1998). The physiology of small airways. Am J Respir Crit Care Med.

[6] Gustafsson PM, De Jong PA, Tiddens HA, Lindblad A (2008). Multiple-breath inert gas washout and spirometry versus structural lung disease in cystic fibrosis. Thorax..

[7] Houltz B, Green K, Lindblad A, Singer F, Robinson P, Nielsen K, et al. Tidal N2 washout ventilation inhomogeneity indices in a reference population aged 7-70 years. Eur Respir J 2012;40(Suppl 56):P3797.

[8] Fuchs SI, Gappa M (2011). Lung clearance index: clinical and research applications in children. Paediatr Respir Rev.

[9] Fuchs SI, Eder J, Ellemunter H, Gappa M (2009). Lung clearance index: normal values, repeatability, and reproducibility in healthy children and adolescents. Pediatr Pulmonol.

[10] Robinson PD, Latzin P, Verbanck S, Hall GL, Horsley A, Gappa M (2013). Consensus statement for inert gas washout measurement using multiple- and single- breath tests. Eur Respir J.

[11] Graham BL, Brusasco V, Burgos F, Cooper BG, Jensen R, Kendrick A, et al. 2017 ERS/ATS standards for single-breath carbon monoxide uptake in the lung. Eur Respir J. 2017;49(1):1600016. 10.1183/13993003.00016-2016 28049168

[12] Poncin W, Singer F, Aubriot AS, Lebecque P (2017). Agreement between multiple-breath nitrogen washout systems in children and adults. J Cyst Fibros.

[13] Stahl M, Joachim C, Wielputz MO, Mall MA (2019). Comparison of lung clearance index determined by washout of N2 and SF6 in infants and preschool children with cystic fibrosis. J Cyst Fibros.

[14] Aurora P, Kozlowska W, Stocks J (2005). Gas mixing efficiency from birth to adulthood measured by multiple-breath washout. Respir Physiol Neurobiol.

[15] Lum S, Stocks J, Stanojevic S, Wade A, Robinson P, Gustafsson P (2013). Age and height dependence of lung clearance index and functional residual capacity. Eur Respir J.

[16] Stanojevic S, Graham BL, Cooper BG, Thompson BR, Carter KW, Francis RW, et al. Official ERS technical standards: Global Lung Function Initiative reference values for the carbon monoxide transfer factor for Caucasians. Eur Respir J. 2017;50(3):1700010. 10.1183/13993003.00010-2017 28893868

[17] Quanjer PH, Stanojevic S, Cole TJ, Baur X, Hall GL, Culver BH (2012). Multi-ethnic reference values for spirometry for the 3-95-yr age range: the global lung function 2012 equations. Eur Respir J.

[18] Nyilas S, Schreder T, Singer F, Poellinger A, Geiser TK, Latzin P (2018). Multiple breath washout: a new and promising lung function test for patients with idiopathic pulmonary fibrosis. Respirology..

[19] Aurora P, Stanojevic S, Wade A, Oliver C, Kozlowska W, Lum S (2011). Lung clearance index at 4 years predicts subsequent lung function in children with cystic fibrosis. Am J Respir Crit Care Med.

